# Diet interventions for depression: Review and recommendations for practice

**DOI:** 10.1177/00048674241289010

**Published:** 2024-12-04

**Authors:** Heidi M Staudacher, Scott Teasdale, Caitlin Cowan, Rachelle Opie, Felice N Jacka, Tetyana Rocks

**Affiliations:** 1 Food & Mood Centre, Institute for Mental and Physical Health and Clinical Translation (IMPACT), School of Medicine, Deakin University, Geelong, VIC, Australia; 2 Discipline of Psychiatry and Mental Health, School of Psychiatry, University of New South Wales, Sydney, NSW, Australia; 3 School of Psychology and Brain and Mind Centre, The University of Sydney, Sydney, NSW, Australia; 4 Food for Thought Nutrition and Dietetics, Glen Iris, VIC, Australia; 5 Centre for Adolescent Health, Murdoch Children’s Research Institute, Parkville, VIC, Australia

**Keywords:** Mediterranean diet, DASH diet, anti-inflammatory, depression

## Abstract

**Objective:**

this paper aims to present the evidence for the role of diet in the prevention and treatment of depression, review the potential underlying mechanisms and provide practice recommendations for mental health clinicians.

**Methods:**

A literature review was conducted through searches of PubMed with the search terms ‘depression’, ‘diet’, ‘prevention’, ‘treatment’ and ‘mechanisms’ and combinations thereof. Additional articles were identified through hand searching.

**Results:**

Greater adherence to several healthy dietary patterns, traditional diets such as the Mediterranean diet and other diets such as the DASH diet are associated with or can treat symptoms of depression. Several limitations of the research were noted, many of which relate to inherent challenges of studying diet. Mechanisms by which dietary intervention can influence mood include the gut microbiome, modulation of inflammatory processes, reduction in oxidative stress and modulation of hypothalamic-pituitary-adrenal axis function. Recommendations for mental health clinicians to enable translation of the evidence into practice are provided.

**Conclusion:**

Diet can play an important role in preventing and treating depression. Mental health clinicians are well placed to provide dietary counselling and to use clinical judgement in choosing the specific approach that reflects the needs of the patient but are encouraged to refer to a specialist dietitian where necessary.

## Introduction

Growing evidence indicates diet can play an important role in the prevention and/or treatment of depression. The exact interplay of mechanisms by which diet can influence mood is unclear; however, the gut microbiome is probably involved. Here, we describe the diets and dietary patterns that have been evaluated in research, discuss the range of mechanisms potentially underlying their effect and critically evaluate the studies that test the effects of dietary interventions on mental health symptoms. Finally, we provide recommendations for mental health clinicians to enable translation of the evidence into practice.

## Diet patterns and depression

A range of diets and dietary patterns have been studied for their potential role in the prevention or treatment of depressive symptoms. The following sections provide a description of each of these diets together with methods for measuring adherence to that diet pattern, both for research purposes and for practice where available. Table 1 provides specific detail about the composition of the major diets covered in this paper.

**Table 1.** Major food group serve recommendations for diets that have been evaluated for their role in improving symptoms of depression.

	Food group	Healthy diet	Mediterranean diet	DASH diet
**Daily Serves**	Fruit	2	⩾3	4–5
	Vegetables	2½–6	⩾2	3–5
	Wholegrains	3–6	Serves not specified but high intake encouraged.	6–8
	Dairy	2–3	Serves not specified but moderate intake encouraged.	2–3
	Olive oil or unsaturated fats	0.5–2 tbs	2–3 tbs	0.4–0.6 tbs
	Combined protein/(e.g. meat, fish, poultry, meat alternatives)	2–3	-	⩽2
**Weekly Serves**	Nuts and seeds	-	⩾3	3–5
	Legumes	-	⩾3	-
	Fish	-	⩾3	-
	Poultry	-	2–3	-
	Red meat	-	⩽1	-
	Alcohol	⩽10–14	⩾7	-
	Extras	0–3	⩽2	⩽5

Data presented for the healthy diet is based on national dietary guidelines for the United Kingdom (British Nutrition Foundation, 2015), the United States (US Department of Agriculture [USDA], 2020) and Australia (AUS), for the Mediterranean diet from the seminal PREDIMED trial (Estruch et al., 2013), for the DASH diet as outlined in the dietary guidelines for the United States (USDA, 2020). Recommended food group servings vary for a healthy diet based on nutrient needs based on age, gender, activity level and weight. Portion sizes varied across all three diets (e.g. one serve fruit = 80 g fresh fruit in the United States, 150 g or one medium piece in AUS).

### Healthy diet pattern

A ‘healthy’ diet typically aligns with national dietary guidelines. These guidelines are founded on scientific evidence about nutrients essential to health, based on intake required to prevent nutritional deficiency and reduce risk of chronic disease, and are targeted to healthy individuals and those with common health conditions (e.g. overweight) but not for people requiring dietary advice for a specific medical condition. Although there are variations in the guidelines across countries, the universal messages are inclusion of a wide variety of vegetables, fruit, wholegrains and legumes and reduction in intake of foods high in saturated fat, added salt or added sugar (Herforth et al., 2019).

In the research context, the ‘healthiness’ of a person’s diet is often rated using a diet quality index (e.g. Healthy Eating Index), which is validated in the target population (Reedy et al., 2018). The diet quality score is usually computed based on dietary data collected using a food frequency questionnaire or diet record. Diet quality component scores are calculated, which are then summed to a total score with a higher score representing a higher quality diet. In practice, diet quality tests are available online and may be useful for mental health clinicians for screening individual’s diets (University of Newcastle, 2021; Commonwealth Scientific and Industrial Research Organisation [CSIRO], 2021).

### The Mediterranean diet and other traditional diets

The Mediterranean diet is an extensively researched traditional dietary pattern shown to have wide-ranging health benefits including a reduced risk for non-communicable diseases (e.g. cardiovascular disease, neurodegenerative diseases, cancer and diabetes) (Dinu et al., 2018; Martinez-Lacoba et al., 2018). The dietary pattern originated from nations in the Mediterranean regions including Greece, Cyprus, Spain and Southern Italy and, as a result, there are several variants based on the local food availability, preferences, and culture within each region (Trichopoulou and Lagiou, 1997). The traditional Cretan Mediterranean diet was the first diet variant to be established as protective for health and formed the basis for the principles of a traditional Mediterranean diet. The diet is characterised by a high intake of vegetables, legumes, fruit, wholegrains, nuts and seeds, a moderate-to-high intake of fish, a low intake of meat, olive oil as the principal source of added fat, and moderate consumption of wine with meals.

Other behavioural and lifestyle recommendations are central to the Mediterranean approach, including eating slowly without distraction, cooking at home, sharing meals with others, incorporating seasonal and local produce and including regular physical activity (Fundación Dieta Mediterránea, 2022). Successful adoption of the Mediterranean diet to non-Mediterranean populations is possible, and studies report take up of the diet into non-Mediterranean communities using an intensive dietitian-led approach incorporating regular support and written resources (Murphy et al., 2022). Challenges such as accessibility to Mediterranean foods, cultural barriers, religion and cost may be prohibitive of adoption and/or long-term adherence in some individuals. While cultural adaptation of the Mediterranean diet may enhance adherence at a population level, it is unknown if a modified Mediterranean diet approach leads to disease prevention to the same extent as a traditional approach.

A range of validated scores have been used to measure adherence to a Mediterranean diet in the published literature. Several scoring systems are recommended for use in research and practice (Hutchins-Wiese et al., 2021), which include relatively brief validated questionnaires such as the MDSS (Monteagudo et al., 2015) and the MEDAS (Schröder et al., 2011).

Other traditional dietary patterns with growing evidence for health benefits include the Nordic diet, traditional Brazilian diet and traditional Japanese diet/Okinawa diet. The Nordic diet has similar ‘plant-based’ principles to the Mediterranean diet but encourages rapeseed (canola) oil instead of olive oil. The traditional Brazilian diet has soy or corn oil as the predominant oil and includes rice and beans in main meals, with small portions of red meat. The traditional Japanese diet is rich in rice, vegetables, soy products (e.g. soybeans and tofu), seafoods and fermented products such as miso and nattō.

### DASH diet

The DASH diet refers to ‘Dietary approaches to Stop Hypertension’. The DASH diet is a healthy diet pattern originally developed for the treatment of hypertension and has demonstrated benefits for reducing the risk of cardiovascular disease (Chiavaroli et al., 2019). It encourages low salt intake and increased intake of foods rich in potassium, calcium and magnesium and emphasises intake of fruit, vegetables, wholegrains, nuts, seeds, legumes, low or no fat dairy products and lean meat.

### Anti-inflammatory diet

Many chronic, non-communicable diseases, including depression, are associated with chronic systemic inflammation. The exploration of known sources of inflammation including psychosocial stress, poor diet and physical inactivity, suggest that inflammation at least partly mediates the path for risk and progression of these chronic diseases (Berk et al., 2013). Intake of a range of specific foods, nutrients and dietary constituents have been associated with lower levels of systemic inflammatory markers and have been described as having anti-inflammatory potential. For example, some studies show adherence to a Mediterranean-style diet, vegetarian/vegan and DASH diet have been associated with lower levels of systemic inflammation (Koelman et al., 2022). Conversely, increased intake of foods and nutrients characteristic of a Western-style diet, including foods high in sugar or saturated fat, can increase inflammatory markers (Christ et al., 2019). The Dietary inflammation Index (DII) is a literature-derived tool that attempts to encapsulate these disparate data (Shivappa et al., 2014). Scores are calculated based on intake of dietary components that negatively and positively contribute to the inflammation score. Dietary components that contribute positively to the total score include fat and carbohydrate, bioactive components such as flavanols, and foods such as ginger, garlic and onion.

## Mechanisms: how could diet influence depression?

The relationship between diet and mental health is multifaceted and complex. Studies examining the underlying mechanisms are still limited and much of the mechanistic research has been conducted in animal models or restricted to the examination of specific dietary components (e.g., micronutrients) and their impact on human health. However, there are several strong candidate mechanisms through which diet influences mental health.

### Gut microbiome

**Figure 1.**
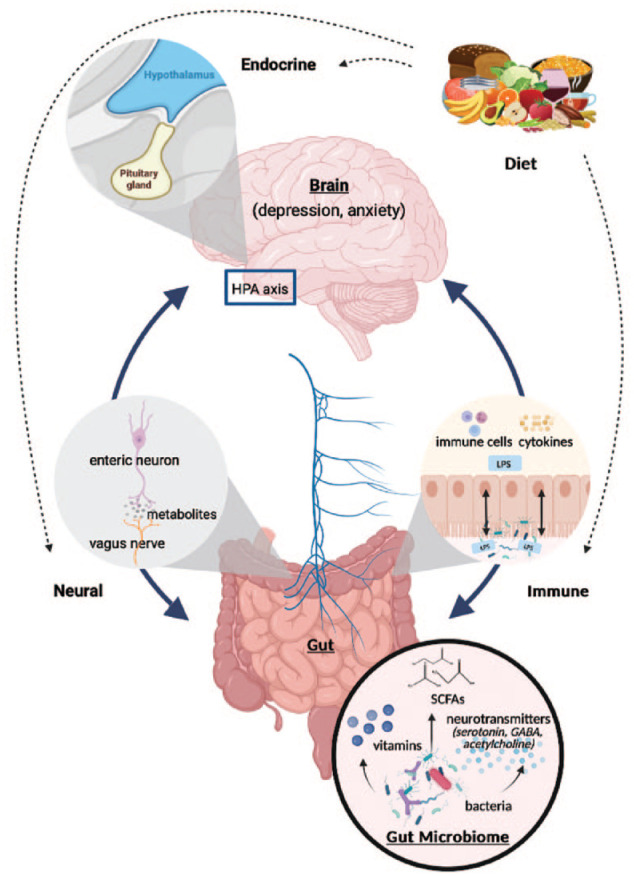
The major pathways through which the microbiome interacts with the brain.

The community of microorganisms within the gastrointestinal tract, known as the gut microbiome, is critical to human health. In particular, the gut microbiome plays an important role in human digestion, nutrient extraction and, throughout this process, direct production or stimulation of production of bioactive metabolites (Rowland et al., 2018). These include short-chain fatty acids (SCFAs), vitamins and several neuromodulators (e.g. serotonin, gamma-aminobutyric acid, catecholamines and acetylcholine; Wiley et al., 2020). Microbial production of neuroactive metabolites represents just one pathway in the complex set of bidirectional lines of communication between the microbiome and the brain, known as the microbiome-gut-brain axis, which includes the vagus nerve, intestinal barrier and the immune system (Figure 1).

Many studies have demonstrated that the microbiome is altered in individuals with clinical depression, and some show abundances of certain microbiome members (or ‘taxa’) are correlated with symptom severity (McGuinness et al., 2022; Simpson et al., 2021). For example, there is consistent upregulation of several taxa with inflammatory properties, such as the opportunistic pathogen *Eggerthella* (McGuinness et al., 2022; Nikolova et al., 2021; Simpson et al., 2021). Other studies identify downregulation of taxa with anti-inflammatory properties or SCFA-producing capabilities in anxiety and depression (McGuinness et al., 2022; Nikolova et al., 2021; Simpson et al., 2021). SCFAs, such as butyrate, are produced through microbial fermentation of indigestible carbohydrates (i.e. dietary fibre) and are involved in a range of pathways within the microbiome–gut–brain axis, including modulation of intestinal barrier and immune function (O’Riordan et al., 2022).

The microbiome is highly responsive to changes in diet. In animal studies, whole diet interventions, macronutrient and micronutrient supplementation have led to changes in both the microbiome and behavioural outcomes (Bear et al., 2020). supporting the idea that the microbiome may mediate diet-induced changes in behaviour. While studies of extreme diet alteration in humans are relatively scarce, significant changes in the microbiome are possible within a single day (David et al., 2014). Mediterranean diet interventions have had limited success in shifting the microbiome, however this may be partly explained by inconsistent diet adherence and variability in baseline microbiome profile (Kimble et al., 2023). A 3-month randomised controlled trial (RCT) in 106 people with obesity found 16 g/d prebiotic inulin supplementation with a high fibre diet had limited effects on mood (Leyrolle et al., 2021). However, exploratory analysis of the treatment responders (i.e. those who reported improved mood following prebiotics) indicated that these individuals exhibited an inflammatory profile and elevated faecal *Coprococcus* abundance at baseline (Leyrolle et al., 2021). These data further support the role of the microbiome in diet-induced mood improvement.

### Inflammation

Immune activation and associated inflammatory processes have an essential role in maintaining physical health in response to injury or infection. However, psychological stress also elicits an inflammatory response, including elevation of cytokines (Steptoe et al., 2007), and inflammatory processes can influence mood and psychological function (Berk et al., 2013). Multiple lines of evidence support the importance of inflammation in the pathophysiology of depression and anxiety in humans. For example, inflammatory cytokines are commonly elevated in individuals with depression and anxiety (Costello et al., 2019; Osimo et al., 2020), cytokine concentration is associated with treatment response in both anxiety and depression (Hou et al., 2019; Strawbridge et al., 2015), and anti-inflammatory treatments can reduce depressive symptoms (Köhler-Forsberg et al., 2019).

**Figure 2.**
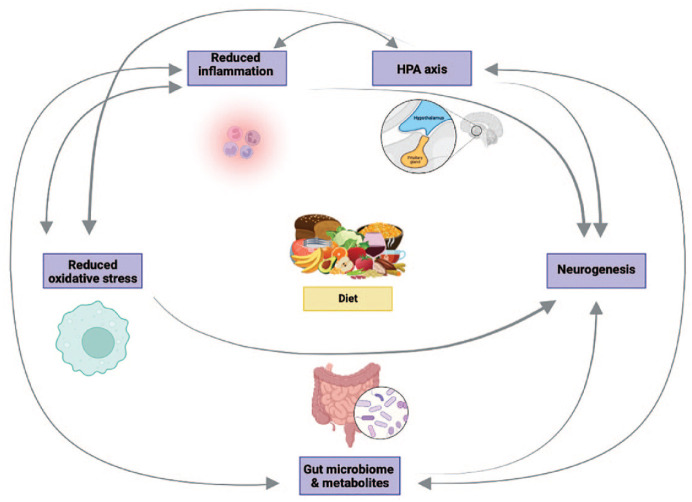
Major candidate mechanisms through which diet can impact mood.

From a dietary point of view, several nutrients have anti- or pro-inflammatory effects. For example, omega-3 fatty acids naturally occurring in oily fish and polyphenols present in blueberries have anti-inflammatory properties (Giacobbe et al., 2020; Yahfoufi et al., 2018). A diet rich in refined carbohydrates and trans saturated fats but low in plant foods is considered pro-inflammatory and is associated with current and future risk of depression (Firth et al., 2018; Lassale et al., 2019). Conversely, the Mediterranean diet has been shown to reduce inflammation (Itsiopoulos et al., 2022), which likely interacts with other postulated mechanisms to influence mental health (Figure 2).

### Hypothalamic–pituitary–adrenal axis

The hypothalamic–pituitary–adrenal (HPA) axis is stimulated in response to threats in healthy individuals, ultimately leading to release of glucocorticoids. Chronic stress and persistent glucocorticoid release can have neurotoxic effects, leading to hippocampal atrophy and dysregulation of the HPA axis (Sapolsky, 2000). Aberrant glucocorticoid responses are observed across a range of psychological conditions (Tafet and Nemeroff, 2020). Importantly, some aspects of HPA axis function are normalised in patients who respond to pharmacological treatment for anxiety and depression (Tafet and Nemeroff, 2020). Food restriction has been shown to dampen HPA axis responsiveness to acute stress in humans and rodents (Rohleder and Kirschbaum, 2007), and specific nutrients such as vitamin C (Brody et al., 2002) and omega-3 fatty acids (Larrieu et al., 2014) can modulate HPA activity. How diet might alter HPA axis signalling remains unclear, but HPA axis involvement in gut-brain signalling suggests the microbiome is involved (Figure 2).

### Oxidative stress

Chronic psychological stress not only disrupts HPA axis function but also promotes oxidative damage, which have consistently been reported in anxiety and depression (Hovatta et al., 2010; Liu et al., 2015). Moreover, successful antidepressant treatment is associated with a reduction in oxidative stress markers (Liu et al., 2015). Specific nutrients with antioxidant properties (e.g. vitamin C, cysteine and polyphenols) have been associated with reductions in oxidative stress in cell culture and animal studies (Zhang and Tsao, 2016). In contrast, a high-fat Western-style diet increases oxidative stress markers in serum and the brain of rodents (Matsuzawa-Nagata et al., 2008; Studzinski et al., 2009). In humans, dietary antioxidant intake has been associated with reduced depression (Ferriani et al., 2022). There is also emerging evidence that antioxidant compounds can have antidepressant and anxiolytic properties in certain clinical populations (e.g. major depression, obsessive compulsive disorder and generalised anxiety disorder; Fernandes et al., 2016; Gautam et al., 2012; Lafleur et al., 2006).

### Tryptophan metabolism

Tryptophan is an essential amino acid that is synthesised into several neuroactive catabolites, including serotonin and kynurenine. Kynurenine production is important because it occurs at the expense of serotonin production, and it is further metabolised into other neuroactive compounds including neuroprotective kynurenic acid and neurotoxic quinolinic acid. A higher kynurenine:tryptophan ratio paired with a decrease in the kynurenic acid:kynurenine ratio is consistently reported in major depression (Marx et al., 2021b) and elevation of quinolinic acid levels has been observed in untreated depression (Ogyu et al., 2018). Tryptophan is derived from a variety of dietary proteins (e.g. meat, fish, nuts, dairy). However, the relationship between diet and plasma tryptophan is complex and manipulating tryptophan levels via dietary means is difficult (Soh and Walter, 2014). Adding to this complexity, tryptophan metabolism is related to several other mechanisms previously discussed, with the microbiome playing a key role in tryptophan degradation.

### Brain-derived neurotrophic factor and neurogenesis

Brain-derived neurotrophic factor (BDNF), the most abundant neurotrophin in the brain, is essential for the growth, differentiation, maintenance and survival of neurons. Supporting its role in the pathophysiology of depression, lower serum BDNF concentrations have been reported in many studies of patients with major depression (Çakici et al., 2020) and increased expression of BDNF has been implicated as one of the mechanisms underlying antidepressant drugs (Castrén and Monteggia, 2021). Intriguingly, specific dietary components, including polyphenols, preserve hippocampal neurogenesis in animal models, whereas a Western diet impairs neurogenesis and lowers hippocampal BDNF (Marx et al., 2021a). A healthy diet is associated with larger hippocampal volume in humans (Jacka et al., 2015) and data from the PREDIMED trial has shown a Mediterranean-style diet with 30 g of nuts a day led to higher circulating BDNF compared with controls (Sánchez-Villegas et al., 2011). The mechanisms by which diet influences neurogenesis are likely through direct and indirect pathways (i.e. via the microbiome; Marx et al., 2021a) (Figure 2).

### Obesity

The relationship between depression and obesity is complex. There appears to be a reciprocal relationship between the two conditions, that is, presence of depression increases risk of obesity, and vice versa (Luppino et al., 2010), however this research tends to rely on body mass index to define obesity, which fails to measure body composition, adiposity or physical fitness. A series of clinical, neurobiological and genetic characteristics, including higher levels of inflammation and related cytokines, are shared between the two conditions, however the nature of the association between obesity and mental disorders remains poorly understood. Importantly, negative attitudes and weight stigma can exacerbate mental health disorders, interfere with positive lifestyle change and contribute to weight gain (Lambert et al., 2019). Considering depressive symptoms can be alleviated by dietary intervention in the absence of weight change (Jacka et al., 2017), the goal of dietary intervention should be to improve diet quality rather than addressing obesity or overweight in people with depression.

## Evidence for diet interventions in treating depression

### Healthy diet

There is a growing body of evidence demonstrating that a ‘healthy’ diet can reduce the risk or treat symptoms of depression. A 2021 synthesis of prospective observational research identified adherence to a ‘healthy’ diet was associated with a reduced risk of depression. This included evidence from 12 studies and over 150,000 individuals (Xu et al., 2021). The strength of the evidence from these studies needs to be interpreted in the light of some challenges. The ‘healthy diet’ defined in studies was heterogeneous and depended on the background preferences of each population. Most studies make appropriate adjustment for confounders (e.g. physical activity, smoking); however, many studies did not control sufficiently for other potential confounders.

Clinical trials provide important evidence that a healthy diet intervention improves depressive symptomatology. In a recent review, 16 trials including a total of nearly 46,000 individuals evaluated the effect of increasing the healthiness of the diet on depressive symptoms (Firth et al., 2019a). Most trials included participants with nonclinical depression and comorbidity such elevated cholesterol, and only one study included a clinically depressed group. Across most studies, diet advice provided was in line with healthy eating principles. Overall, there was a small effect of healthy eating interventions on reducing depressive symptoms. Interestingly, those studies that incorporated advice from registered nutrition professionals were most beneficial. Based on the totality of the evidence, healthy eating advice is beginning to be incorporated into clinical and lifestyle guidelines for mood disorders (Malhi et al., 2018; Marx et al., 2023).

### Mediterranean diet

The Mediterranean diet has a growing evidence base for the prevention and treatment of depressive disorders. The pooled effect of four longitudinal studies with a mean follow-up period of approximately 10 years found a 33% risk reduction in the development of depression for the highest compared to the lowest adherence categories (Lassale et al., 2019). Recently, a large multi-centre 2 × 2 factorial design 1-year RCT (*n* = 1025) evaluated whether modifying a person’s diet to be more aligned with the Mediterranean diet was effective in preventing the development of a depressive disorder. The intervention group received food-related behavioural activation therapy, conducted by a psychologist, incorporating the Mediterranean diet and multi-nutrient supplementation (together and in isolation) and the control groups received placebo and no therapy (Bot et al., 2019). Although there was no difference in incident depression between groups in the primary analysis, after accounting for food-related behavioural activation therapy adherence, there was a significant favourable effect of food-related behavioural activation on incident depression, indicating further research is required to assist understanding of the role of diet in prevention of depression (Bot et al., 2019b).

Stronger and more consistent evidence has been found for Mediterranean diet intervention to treat symptoms in people with clinical depression (Bayes et al., 2022; Francis et al., 2019; Jacka et al., 2017; Parletta et al., 2019). The 2017 ‘SMILES’ 12-week parallel group (*n* = 56 adults) trial found a dietitian-delivered, modified Mediterranean diet intervention was superior to a social support group in reducing depressive symptoms, with remission being achieved in 32% of the Mediterranean diet group compared to 8% in the social support group (Jacka et al., 2017). The 6-month ‘HELFIMED’ trial (*n* = 152 adults), incorporating nutrition education and fish oil supplementation demonstrated similar findings (Parletta et al., 2019). In both trials, improvements in Mediterranean diet adherence scores aligned with reductions in depressive symptoms (Opie et al., 2018; Parletta et al., 2019). Similar findings have been reported in younger individuals, including a 12-week RCT (*n* = 72) in young males (Bayes et al., 2022), and a 3-week RCT in young adults (17–35 years) (Francis et al., 2019), both finding a greater reduction in depressive symptoms in the Mediterranean diet group.

Considering the frequent comorbidity of anxiety in individuals with depression, it is interesting to note there is reasonable consistency in the observational evidence for the Mediterranean diet being protective against anxiety (Madani et al., 2022), however intervention studies in people with anxiety disorders are lacking.

### Other traditional diets

The beneficial effect of traditional diet interventions on depression may not be limited to the Mediterranean diet, however there is less evidence for other traditional dietary patterns. For example, a cross-sectional study of Japanese men and women aged 21–67 years (*n* = 521), found a 56% risk reduction for depressive symptoms in those with the highest adherence compared with the lowest adherence (Nanri et al., 2010). In a 12-week RCT, of people with severe obesity in Brazil (*n* = 149) were randomised to 52 mL/day of extra virgin olive oil, traditional Brazilian diet, or traditional Brazilian diet with 52 mL/d of extra virgin olive oil. There was a significant reduction in depression and anxiety scores in all three groups (Canheta et al., 2021). Finally, a small RCT (*n* = 16) has reported feasibility of a Nordic diet as a treatment for depression (Sabet et al., 2021), providing a platform for larger efficacy trials.

### DASH diet

The DASH diet has predominantly been studied in the context of hypertension and cardiovascular disease and is recommended in clinical guidelines for prevention of cardiovascular disease (Chiavaroli et al., 2019). The overlapping pathophysiologies of hypertension and depression (e.g. hyperactivation of the HPA axis, oxidative stress, low grade inflammation) spurred interest in whether the DASH diet might also have a protective role for mood disorders. A large prospective study of the Spanish SUN cohort showed a moderate relationship between four DASH diet indices and depression risk over 8 years of follow-up (Perez-Cornago et al., 2017), in contrast with the clearer inverse relationship between the Mediterranean dietary pattern and depression in the same cohort (Sánchez-Villegas et al., 2009). Only one RCT to date has studied the DASH diet and its effect on depressive symptoms, which failed to show an effect on depressive scores compared with a healthy diet control group that had lower targets for fruit and vegetable intake (Torres and Nowson, 2012). Overall, to date there is insufficient evidence supporting a DASH diet for the prevention or treatment of depression.

### Anti-inflammatory diet

People with depressive disorders appear to have dietary intakes with higher inflammatory potential based on DII scoring. An analysis of 68,879 people from the baseline phase of the UK Biobank study (2007–2010), found DII scores were significantly higher for people with major depression compared to people without a serious mental illness, after adjusting for a range of confounders (Firth et al., 2018). Evidence from 11 observational studies (*n* > 100,000) also reports a pro-inflammatory diet is associated with 1.4 times higher odds of having/developing depression in the future (Tolkien et al., 2019).

### Limitations of the research

There are a range of limitations of the research evaluating the role of diet intervention for treating or preventing depressive symptoms. A considerable proportion of RCTs have been conducted either in general population samples or clinical samples with a primary physical health condition, rather than in samples of people with a clinical diagnosis of a depressive disorder. Where individuals with a depression diagnosis are included, participant selection occurs via self-reported depression questionnaires rather than a clinical diagnosis, meaning findings are not necessarily generalisable to those with a clinical disorder. Response bias may also be significant in studies due to involvement of participants with strong interest in diet. Interventions may therefore appear feasible and effective based on data from RCTs but are less promising when scaled up to reach a larger proportion of the clinical population.

Other factors challenge synthesis and interpretation of studies. There is large heterogeneity of instruments used to measure depressive symptoms as well as dietary intake and adherence. Particularly relevant for observational studies, many measure dietary intake at a single timepoint which fails to capture temporal variability in eating behaviour. Diet RCTs differ in the mode of delivery of diet intervention, the nature of the intervention (e.g. some include counselling and goal-setting), the presence of blinding and the nature of the control intervention. Finally, many intervention studies are short-term and lack long-term follow-up. Therefore, the ability of individuals to maintain adherence and the long-term effectiveness of diet interventions remains unclear.

### Recommendations for future research

Enhancing diet quality is one of a suite of lifestyle changes that can improve symptoms of depression. Future trials must explore whether combined intervention models (e.g. diet + exercise intervention) are superior and also disentangle the effects of individual lifestyle components. Further to this, the efficacy of dietary modification as a sole intervention, given published studies rarely restrict the use of psychotropic medications, is still to be confirmed. The literature is also biased towards individuals from high-income countries, and the role of dietary modification in lower-income countries where food insecurity rates are considerably high and access to more traditional mental health care are already strained needs to be determined.

Symptoms central to depressive disorders can often challenge recruitment to studies as well as engagement and adherence to behavioural interventions. Recruitment strategies should therefore aim to include a diverse range of participants, or at least clearly define the sample group when publishing findings.

The heterogeneity and generalisability of diet intervention delivery needs consideration. Some elements (e.g. provision of food hampers) are not feasible when scaled to real world implementation. Furthermore, a better understanding of the nature and intensity/frequency of counselling, and efficacy of different delivery modes (e.g. face-to-face individualised, group) is required. Ideally, future trials should evaluate modes of delivery that are easily adopted and delivered at scale, and mode of delivery and diet composition should be clearly specified.

Individuals with depression are a heterogeneous population. Patients experience a variety of symptoms and levels of acuity, with the potential for differing triggers, different treatment approaches and associated comorbidities. This is an important consideration as the efficacy of different treatment approaches may differ across subgroups, for example, reducing inflammation may only be beneficial to the subgroup of people with depression who have elevated inflammatory markers. Identification of specific biopsychosocial predictors to diet intervention will be an important step forward.

## Application to practice for mental health clinicians

### Dietary assessment and counselling

Dietitians are nutrition experts trained in providing individualised dietary advice for health and disease. A range of clinical, physical, mental and social factors are considered throughout the nutrition care process, as many of these are critical for successful patient engagement. Commonly, the nutrition care process includes assessment of dietary intake, assessment of the patient’s knowledge and motivation to change, discussion and identification of the issues and barriers to be addressed, provision of information, nutrition counselling and evaluation of dietary changes that have previously been implemented. Although many individuals will benefit from dietary assessment and/or counselling by their mental health clinician, there are some individuals that should be referred to a dietitian, such as those with a history of or existing eating disorders (e.g. anorexia nervosa, bulimia nervosa) or those with disordered eating. In those with disordered eating, individuals may experience symptoms and exhibit behaviours of eating disorders (e.g. inflexible eating patterns, restrictive eating or dieting), but at a lesser frequency or lower level of severity. The prevalence of disordered eating is up to 32% in younger individuals and is more prevalent in those with mental health problems (Sparti et al., 2019). Individuals who are young, pregnant or elderly, and those with additional nutrition needs (e.g. comorbid disorders such as gastrointestinal disorders or type 2 diabetes) or with loss of appetite or significant weight change should also be referred to a dietitian for their nutrition care.

An important first step in the nutrition care process for any clinician seeing individuals with depression is the assessment of dietary intake and dietary behaviour. For a clinician not trained in nutritional assessment techniques, this could start with simple open questions regarding how many meals and/or snacks are eaten per day, if the patient cooks for themselves, or whether they can describe a typical day’s intake. Initiating a discussion around food and eating will also help to provide some understanding about an individual’s knowledge about nutrition and its importance to the individual.

Second, identifying the main barriers for healthy eating is needed to construct a patient-centred plan and to identify the support required to achieve their nutrition goals. The typical side-effects of common mental disorders should be considered (e.g. altered appetite [reduced, increased or food-specific cravings], apathy, low motivation, impaired concentration and memory) when assessing these barriers.

Third, to facilitate dietary behaviour change, psychoeducation and evidence-based resources should be provided. Clinicians are recommended to provide diet counselling in a sensitive manner, cognisant that many individuals experience shame and guilt when discussing weight and diet behaviours. Dietary information should be delivered in accessible, bite-size format, and take into consideration the individuals learning styles, baseline knowledge and beliefs, motivations, readiness to engage in sessions, and relevant lifestyle factors (e.g. work commitments, family dynamic). Importantly, motivation and readiness to change can be highly variable and should be assessed in each session. When the individual is ready, focusing on small, attainable and value-based goals will build motivation and self-capacity for change. For example, providing practical tips for cooking and preparing meals and snacks may be warranted, with a large emphasis on forward planning (e.g. developing shopping lists, preparing meals in advance and stocking the home with long shelf-life foods) for times when a patient is experiencing increased severity of symptoms. To support dietary adherence, recommendations should also align with common cravings. For example, nutritious foods can be encouraged that address cravings for ‘sugary’ foods (e.g. Greek yoghurt with walnuts, honey, fruit) and ‘salty’ ‘fatty’ foods (e.g. olives and nuts).

Finally, through reflective practice, evaluating outcomes and identifying helpful strategies that have facilitated change will assist the clinician in fine tuning their dietary assessment and counselling skills (Koshy et al., 2017).

### Clinical considerations

Individuals with depression commonly present with other psychological comorbidities (e.g. anxiety) and chronic physical conditions (e.g. cardiovascular disease, metabolic syndrome and type 2 diabetes). Many of these are responsive to dietary intervention. There is strong evidence that diet interventions for depression (i.e. Mediterranean diet and DASH diet) also achieve improved health outcomes at least for cardiovascular disease and diabetes (Martinez-Lacoba et al., 2018), and therefore there is potential for dietary counselling to address both mental and physical health conditions in the same individual. In any case, a weight-neutral approach is critical (i.e. lack of focus on weight loss but rather health behaviour change) (Hunger et al., 2020). Weight stigma and negative attitudes about one’s body weight and shape hinder positive changes, creates unnecessary pressure and can promote poor eating behaviour and low motivation (Vartanian and Porter, 2016). Evidence demonstrates that improvements in depressive symptoms (and physiological health) can be achieved with dietary improvement without weight loss and in higher weight people (e.g. body mass index = 30 k/gm^2^) (Jacka et al., 2017).

### Social considerations

Individuals living with mental illness commonly experience high rates of unemployment or missed days at work and therefore may lack adequate finances to access food or to access nutrition services. Food insecurity relates to limited or uncertain access to sufficient, safe and nutritious food for normal growth and development and an active and healthy life (Food and Agriculture Organisation of the United Nations [FAO], 2022). This may include lack of resources, access to nutritious food at an affordable price, access due to geographical isolation, cooking facilities, and knowledge or skills to make appropriate choices. The clinician should ascertain the degree of food insecurity experienced by the individual and modify dietary recommendations accordingly, for example, strategies for preparing meals with minimal storage; free community meals; community cooking classes. For individuals requiring dietetic assessment and counselling, clinicians should explore potential subsidised dietetic services available and how they may access them.

### Service considerations

Dietary consultations should be delivered using an evidence-based approach underpinned by peer-reviewed scientific research, while considering the therapeutic relationship with the patient (Nagy et al., 2022). With regard to the intensity and frequency of nutrition care for efficacy in depression, evidence from the SMILES trial shows that an intervention encompassing seven individualised face-to-face sessions with a dietitian involving motivational interviewing and goal setting, with the aim to encourage dietary change, delivered over a 3-month period (e.g. weekly to fortnightly sessions) can achieve reductions in depressive symptoms (Jacka et al., 2017; Opie et al., 2018). In practice, initial dietetic appointments are of 60 minutes duration and review appointments are of 30–60 minutes duration (Dietitians Australia, 2019). This degree of service frequency and intensity can allow for meaningful changes in dietary quality The complex nature of common mental disorders and the many facets of the individual and their multiple health concerns often necessitate more frequent sessions. Those with lower readiness to change, or limited food-related knowledge or skills, may benefit from a greater number of diet-focused sessions, longer period of extensive support and/or ‘restart’ opportunities. For a long-term effectiveness, diet interventions should be flexible, with continuous motivational support and broad multifaceted strategies for change (Firth et al., 2019b).

### Delivery considerations

Developing a meaningful therapeutic relationship is considered crucial in nutrition practice and pivotal to how effectively the patient and the professional engage and work together (Nagy et al., 2022). Face-to-face consultation allows for tailoring or personalisation of nutrition information to the individuals’ requirements and lifestyle with the aim of facilitating behaviour change. Evidence from RCTs of personalised nutrition interventions demonstrate that compared to web-based interventions, face-to-face nutrition interventions can achieve significantly greater dietary change, and are also more likely to produce long-term benefits (Al-Awadhi et al., 2021). In addition, supporting a patient to attend in-person appointments can play an important role in working through motivation, encouraging forward planning and routine. However, face-to-face nutrition services can be expensive, time-consuming and may not be accessible to everyone (Al-Awadhi et al., 2021).

Following technological advances, web-based and mobile methods of dietary advice are increasingly replacing or supplementing face-to-face consultations. Telehealth offers a convenient service that can be especially desirable for individuals with mood disorders who commonly experience amotivation and/or agoraphobia. In addition, it can reduce the burden associated with regular attendance at appointments and potential financial concerns (e.g. cost of transport). Telehealth and web-applications can reach a larger population and allows patients with mobility issues to access services. Importantly, web-based nutrition interventions should be personalised in order to support dietary change, but there is currently insufficient evidence that web-based interventions are as effective as face-to-face interventions (Al-Awadhi et al., 2021).

It is worth considering the use of multiple delivery modes which allows for development of a therapeutic alliance in the initial phases, while allowing for a convenient service with reduced patient–clinician burden in the latter phase (e.g. initial face-to-face consultation with videoconference review appointments). Finally, group consultations should be considered in the context of providing a valuable opportunity for peer support (Watkins et al., 2020).

### Final considerations

Diet interventions can lead to impressive reductions in depressive symptoms, particularly when advice is dietitian-delivered and with sufficient frequency of sessions. However, psychiatrists, psychologists and other mental health clinicians can play an important role in the assessment and counselling of individuals with regard to diet, and at least raising patient awareness of the role of diet in mental health. For example, clinicians could discuss with patients of the current evidence that healthy dietary patterns may be instrumental in managing symptoms of depression. Conversely, a high intake of discretionary items (e.g. crisps and sugar sweetened beverages) increases the risk of poor mental health. This must be delivered in a highly sensitive non-stigmatising fashion, as patients commonly experience shame around food, eating and body weight. Support should focus on positive behavioural changes (e.g. increasing intake of legumes by one serve/ week) and should be weight-neutral. Raising the profile of nutrition is highly relevant and in accordance with Royal Australian New Zealand College of Psychiatrists clinical practice guidelines stating lifestyle approaches should form the foundation of treatment for mood disorders (Malhi et al., 2018).

Psychiatrists and other members of the mental health team also play a part in working a patient up for a referral to a mental health dietitian. This work-up involves establishing an individual’s interest in dietary change, their motivation level and readiness to change, and presence of potential ‘red-flags’, including eating disorders, disordered eating, poorly managed comorbid medical health conditions, loss of appetite, significant weight change, need for a specialised diet or pregnancy.

## Conclusion

The large burden of early mortality due to cardiometabolic illness, alongside the more recent evidence for diet quality as a risk factor and treatment target for mental health problems, support the imperative for the use of dietary interventions to treat depression. There is particularly compelling data to support Mediterranean diet intervention to improve symptoms of depression. Given the high rates of cardiometabolic comorbidity in individuals with depression, this is a favourable approach because of its protective effects for metabolic syndrome and cardiovascular disease. Mental health clinicians are well placed to provide nutrition counselling and to use clinical judgement in the timing of nutrition counselling and the choice of approach that reflects the needs of the patient but are encouraged to refer to a specialist dietitian where necessary.
